# Optimizing Microbubble Reduction to Facilitate IVUS Guidance During Endovascular Radiofrequency Wire Procedures

**DOI:** 10.3390/tomography12040048

**Published:** 2026-03-31

**Authors:** Curtis Plante, Andrew E. Warfield, Carlos Escobedo, Amer M. Johri, David S. Majdalany, Bill S. Majdalany

**Affiliations:** 1 Division of Interventional Radiology, Department of Radiology, University of Vermont Medical Center, 111 Colchester Ave., Burlington, VT 05401, USA; curtis.plante@med.uvm.edu (C.P.); andrew.warfield@med.uvm.edu (A.E.W.); 2 Department of Chemical Engineering, Queen’s University, 19 Division St, Kingston, ON K7L 2N9, Canada; carlos.escobedo@queensu.ca; 3 Department of Medicine, Queen’s University, 94 Stuart Street, Kingston, ON K7L 3N6, Canada; 4 Department of Cardiovascular Diseases, Mayo Clinic, Rochester, MN 55905, USA

**Keywords:** ultrasound, intravascular ultrasound, microbubble, radiofrequency wire, radiofrequency puncture

## Abstract

**Background/Objectives:** Radiofrequency (RF) wire energy can be used for tissue ablation across many conditions. Adjusting RF generator parameters allows RF energy to puncture tissue with minimal adjacent damage. When RF energy is applied to tissue, however, microbubbles are produced, obstructing intravascular ultrasound (IVUS). Mitigation of RF-generated microbubbles has been studied for ablation but not for puncture. **Methods:** This paper describes an in vitro bench study using ex vivo bovine live tissue. A model was created with bovine liver tissue and an IVUS catheter submerged in a saline bath. Tissue was punctured with an RF guidewire to recreate microbubbles. Following the puncture, various methods were applied: altering the mechanical index of the IVUS, applying a VF10-5 Linear probe (Siemens), and applying a L12-3 Linear probe (Philips). Regions of interest (ROIs) were selected to track pixel brightness as a proxy for microbubbles. **Results:** The control increased ROI brightness by 1.5%. Altering the mechanical index of IVUS reduced ROI brightness by 1.2%. VF10-5 probe application increased ROI brightness by 1.2%. L12-3 probe application reduced ROI brightness by 33.0% (*p* = 0.046, *n* = 3, one-sample *t*-test). Brightness reduction was most pronounced at the site of initial RF wire puncture, where microbubbles accumulated. Tip visualization improved, allowing for more precise wire trajectory adjustments. **Conclusions:** External US with an L12-3 probe was able to dissipate microbubbles effectively to improve IVUS guidance following RF wire puncture in an in vitro exploratory bench model.

## Simple Summary

Radiofrequency wires use energy to puncture dense tissue during medical procedures. When radiofrequency energy is applied, bubbles form that block intravascular ultrasound imaging, making it difficult to see and guide the wire accurately. This study tested different methods to clear these bubbles using a bovine liver tissue model. The most effective approach used an external ultrasound probe, which reduced bubble interference by 33% and improved visualization of the wire tip. These findings suggest that widely available ultrasound equipment could improve guidance during radiofrequency wire procedures, though more testing is needed to confirm effectiveness in patients.

## 1. Introduction

Radiofrequency (RF) energy is commonly used for targeted tissue ablation to treat a variety of conditions, including atrial fibrillation, joint pain, and tumors [1,2,3]. Adjusting RF generator parameters allows an RF wire to puncture tissue. RF puncture applies short bursts of energy to vaporize through tissue while minimizing damage to the surrounding area. This is particularly applicable when puncturing denser tissues that deflect mechanical needles. In some cases, tissue may be too dense to traverse with a mechanical needle, thereby requiring RF puncture techniques [4,5,6,7,8]. When vaporizing tissue, RF energy rapidly produces microbubbles which obstruct RF wire tip visualization on intravascular ultrasound (IVUS) imaging [9,10]. IVUS image guidance continues to be a valuable tool for endovascular procedures. It has been shown to reduce total procedure time, radiation dose, contrast volume, and number of punctures for multiple endovascular procedures [11,12]. However, RF obstruction of IVUS image guidance must be investigated and optimized before the two technologies can be used together widely.

Acoustic disruption via high mechanical index (MI) ultrasound (US) is a safe, reliable, and low-cost method to disrupt microbubbles within the body. Clinically, high-MI US is already widely used to rupture microbubbles in myocardial and hepatic contrast perfusion studies [13,14,15]. These techniques use a flash of high-MI US to break contrast microbubbles and assess perfusion through microbubble replenishment in the imaging field. US is also being studied to improve drug delivery through US-targeted microbubble destruction [16,17,18]. US-targeted microbubble destruction involves the targeted cavitation of drug-containing microbubbles to more effectively deliver chemotherapeutic and immunotherapeutic agents. This protects therapies from endogenous clearance and reduces systemic side effects. Both perfusion studies and targeted drug delivery illustrate the clinical effectiveness of high-MI US to safely and reliably disrupt microbubbles in the body. The demonstrated clinical effectiveness, low cost, and wide availability make high-MI US a suitable candidate for disrupting RF-generated microbubbles.

Modeling and reduction of microbubble production from RF ablation have been previously investigated [19,20,21]. Seil Oh et al. described an “avoiding microbubbles” protocol for pulmonary vein antrum RF ablation [20]. This protocol involves a constant increase in generator wattage with downward power titration on visualization of RF-generated microbubbles. When compared to a traditional temperature-guided ablation protocol, the “avoiding microbubble” protocol reduced the risk of procedural complications during RF energy delivery. Alan Sugrue et al. developed a retractable hood to surround the ablation catheter and filter RF-generated microbubbles as they are produced [21]. This filtration system significantly reduced the number and volume of microbubbles produced during RF ablation. While the described methods are effective at reducing microbubble generation and complications during RF ablation, there are currently no techniques that are feasible for RF puncture. Furthermore, no previous studies have explored microbubble mitigation during RF puncture. This article aims to replicate, study, and evaluate methods to limit microbubble generation and facilitate IVUS image guidance during RF puncture.

## 2. Materials and Methods

### 2.1. Model Construction and Equipment

**Figure 1:**
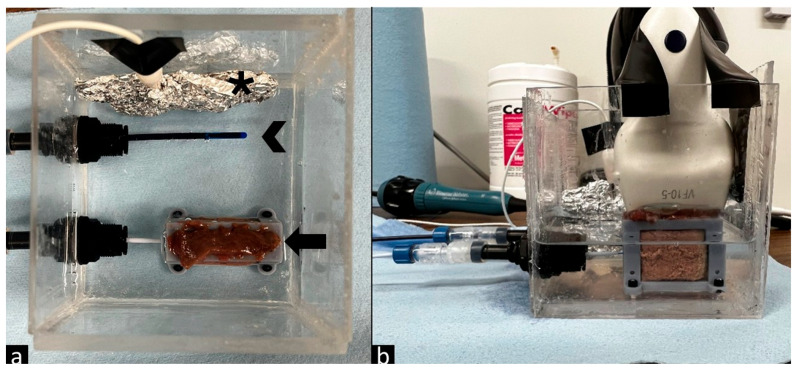
(**a**) Experimental setup top view with return electrode (asterisk), IVUS catheter (arrowhead), and RF wire in line with tissue (arrow). (**b**) Experimental setup side view, including a Siemens VF10-5 probe attached to the exposed liver tissue and secured to the container.

This paper describes an in vitro bench study using ex vivo bovine live tissue. The tissue was acquired from a local butcher/grocer. No alternative, animal care or husbandry was involved. There was no direct harm to animals for the purposes of this study. While suitable for feasibility testing, the use of ex vivo bovine liver tissue limits direct translation of these findings to human clinical settings. A custom experimental model was constructed to securely hold bovine liver tissue in a saline bath and allow for the insertion of both an RF guidewire (PowerWire, Baylis Med Tech, Mississauga, ON, Canada) and an IVUS catheter (Soundstar ultrasound catheter, Biosense Webster, Irvine, CA, USA). These were introduced horizontally to trap bubbles within the liver tissue, as vertical puncture allowed bubbles to escape. Adjustable tube fittings and Tuohy-Borst adapters were used to insert the wires without solution leaking from the container. When external US probes were used, they were secured to the top of the liver tissue prior to any imaging. Figure 1 illustrates the experimental setup and organization of these materials.

Setup construction began with the cutting of acrylic sheets into five 10 cm × 10 cm square pieces: two thin (0.32 cm thick) squares for the front/back and three thick (0.64 cm thick) squares for the base and sides. Then, two holes were drilled through one of the thin sheets with a 16 mm drill bit. These holes were drilled 2 cm above the sheet’s base and 4.5 cm apart. On a thick sheet, four holes were drilled halfway through the acrylic with a 2.38 mm bit and tapped with a #43 tap. These holes were positioned to be in line with one of the previously drilled 16 mm holes (when glued) and to match the screw openings for the designed tissue holder. The edges of each acrylic sheet were then sanded to ensure a flat, bondable surface and bonded to form an open-box container with quick-dry acrylic glue. All edges of the box were sealed with silicone sealant. Once the sealant dried, the tissue holder was attached to the base with four 4–40 thread, plastic screws. Two tube fittings were then tightened in the 16 mm drill holes with rubber O-rings. Silicone connection tubing was cut into two 2.5 cm long pieces and secured around these connectors. Finally, Tuohy-Borst adapters were attached to the end of the silicone tubing; these adapters were then tightened around the RF guidewire support or IVUS catheter to prevent solution from leaking.

Seven pieces of equipment were used in this study: one IVUS catheter, two US systems, two US probes, one RF generator, and one RF wire. Table 1 presents the equipment used with their respective specifications and operational settings. Table 2 presents the materials used during bench setup construction.

### 2.2. Experimental Procedure

The experimental procedure was divided into five stages: initial imaging, RF puncture, midpoint imaging, method application, and final imaging. These divisions allowed for a consistent procedure, which was altered at the application stage to evaluate different proposed methods. For a baseline, the same procedure was used with a two-minute waiting period during the method application stage. New liver tissue and saline solution were used for each experimental trial. Bovine liver tissue was acquired from a local butcher; no live animals were used in this study

First, a piece of bovine liver was secured in the tissue holder and imaged with the IVUS catheter to record an initial picture of the tissue. The liver was then punctured with RF energy for three seconds with the RF generator (RFP-100A RF Puncture Generator, Baylis Medical Company) set to 25 Watts. After removing the wire, the liver was reimaged with the IVUS catheter to view the RF-generated, trapped microbubbles. One of the proposed methods was then applied to the system, and the liver was reimaged to evaluate the method’s ability to dissipate microbubbles. Three methods to reduce RF-generated microbubble obstruction of IVUS imaging were tested: altering the MI of the IVUS catheter, VF10-5 probe (Siemens) application, and L12-3 probe (Philips) application. All were applied over a single, ten-second interval. Superficial US probes were secured to the top of the model before RF puncture.

All experimental trials were conducted by a single operator to limit procedural variability in both RF puncture and US imaging. Due to resource limitations on the number of trials, each method was recorded once, with two additional trials of the L12-3 probe after initial qualitative analysis. This study was designed to explore the feasibility of external US on RF puncture image obstruction and was not powered for definitive inference.

## 3. Results

After RF puncture, IVUS echogenicity was increased by multiple factors, including microbubble production, thermal scarring, and local hemorrhage with coagulation. Tracking IVUS pixel brightness around RF puncture sites was determined to be the most appropriate method for assessing microbubble burden after RF puncture and US application. Therefore, pixel brightness was used as a proxy to measure image obstruction by RF-generated microbubbles, with the understanding that other tissue changes may contribute to increased brightness after RF puncture. A script was written in MATLAB 2022b to manually select a region of interest (ROI) and track average normalized pixel brightness. ROI selection was completed by one operator with standardized polygonal selection around the RF puncture trail to reduce ROI subjectivity. Percent reduction of RF puncture obstruction was calculated as a ratio of the brightness difference before and after method application to the brightness difference before and after RF puncture. This represents the amount of brightness introduced by RF puncture, which was dissipated by the applied method. ROI brightness data, calculated differences across experimental stages, and percent reduction of RF puncture obstruction are reported in Table 3.

**Figure 2:**
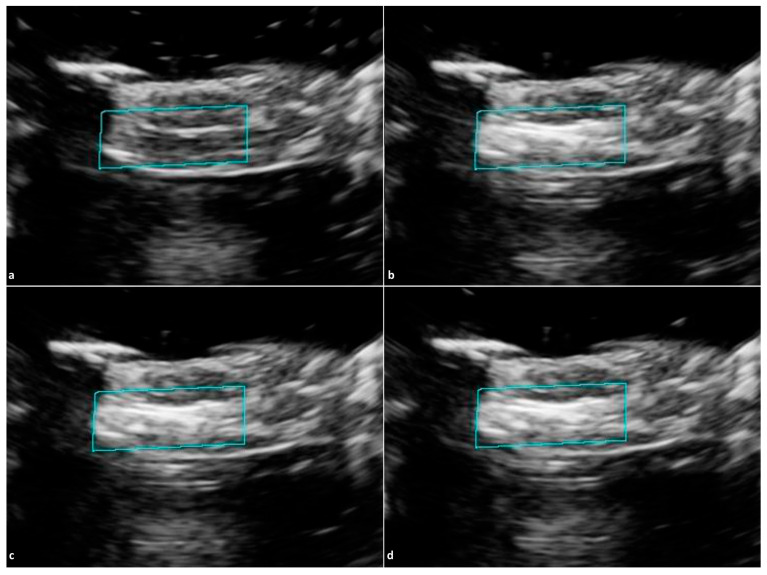
(**a**) Baseline IVUS images with selected ROI (blue-green box) before RF puncture, (**b**) after RF puncture, (**c**) 1 min after RF puncture, and (**d**) 2 min after RF puncture. No application or external influence was introduced to the tissue post-RF puncture.

**Figure 3:**
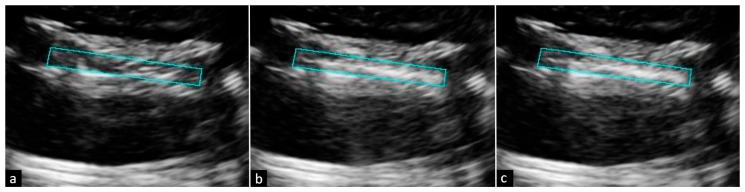
(**a**) IVUS images of the IVUS MI increase method with selected ROI (blue-green box) before RF puncture, (**b**) after RF puncture, and (**c**) after IVUS MI increase. IVUS MI was increased from 0.62 to 0.70 for 10 s before returning to 0.62 for final imaging.

**Figure 4:**
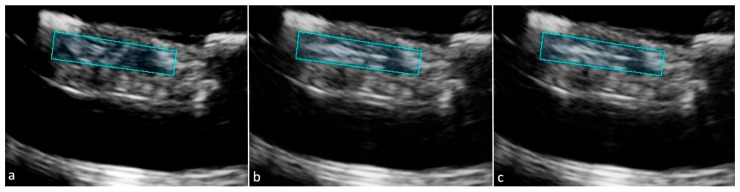
(**a**) IVUS images of the VF10-5 probe application method with selected ROI (blue-green box) before RF puncture, (**b**) after RF puncture, and (**c**) after VF10-5 application. The VF10-5 probe was secured to the top of the model tissue before initial imaging. Following RF puncture, US from the VF10-5 probe was applied for 10 s before reimaging on IVUS.

**Figure 5:**
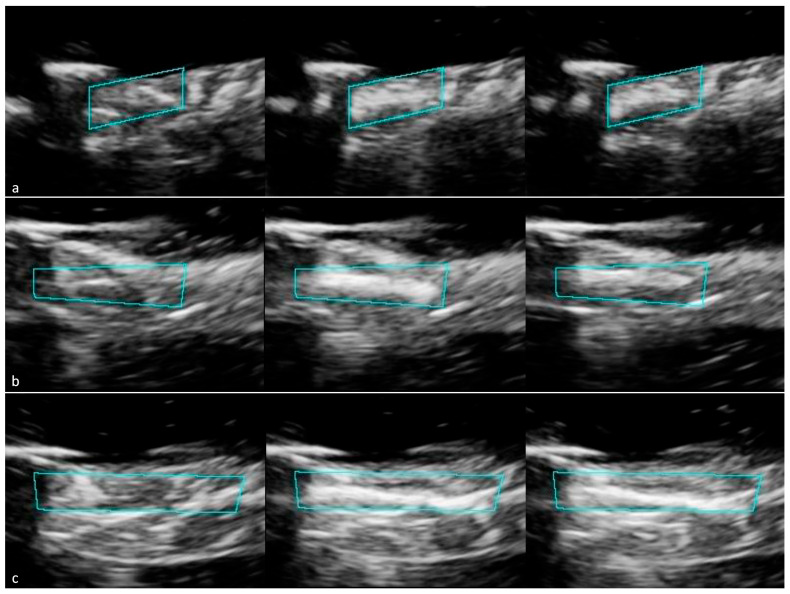
IVUS images of the L12-3 probe application method with selected ROI (blue-green box) for all trials. The L12-3 probe was secured to the top of the model tissue before initial imaging. Following RF puncture, US from the L12-3 probe was applied for 10 s before reimaging on IVUS. (**a**) Trial 1 images-before RF puncture (left), after RF puncture (middle), and after L12-3 application (right). (**b**) Trial 2 images before RF puncture (left), after RF puncture (middle), and after L12-3 application (right). (**c**) Trial 3 images before RF puncture (left), after RF puncture (middle), and after L12-3 application (right).

**Figure 6:**
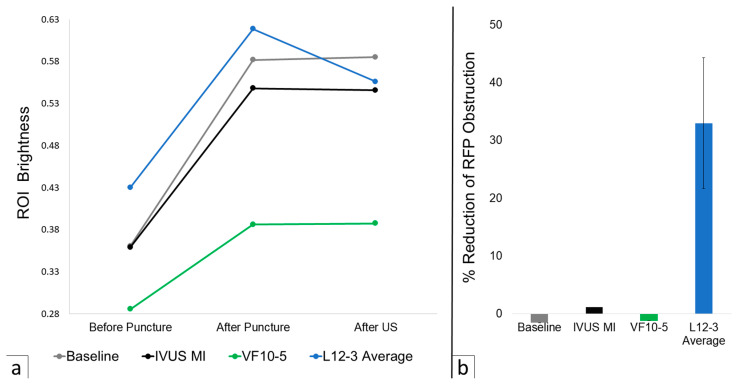
(**a**) Scatterplot of average ROI brightness at each experimental stage for the baseline, IVUS MI increase, VF10-5 application, and L12-3 application average. Note: the baseline data point for ‘After US’ represents after a waiting period of two minutes. (**b**) Bar graph of calculated % reduction of RF puncture obstruction for the baseline, IVUS MI increase, VF10-5 application, and L12-3 application. A SEM error bar is included for the L12-3 average. See Table 3 for raw data and calculated values.

Across all trials, ROI brightness increased by 47.3% on average following RF puncture. The baseline with a two-minute waiting period increased ROI brightness by 1.5%, as seen in Figure 2. Altering the MI of IVUS reduced ROI brightness by 1.2% as seen in Figure 3. VF10-5 probe application increased ROI brightness by 1.2% as seen in Figure 4. Application of the L12-3 probe reduced ROI brightness by 33.0% ± 22.1% (one-sample *t*-test, *p* = 0.046, *n* = 3) as seen in Figure 5. The L12-3 probe showed a statistically significant reduction of ROI brightness on IVUS guidance at the 95% confidence level. This rejects the null hypothesis that ROI brightness decreases by the same amount after L12-3 probe application as after a baseline waiting period of two minutes following RF puncture. Statistical analysis was conducted using Microsoft Excel. A one-sample *t*-test was used to compare methods to a value reported by the baseline trial. This reflects the assumption that after RF puncture, liver tissue echogenicity on IVUS does not change over time without external influence. All data were assumed to be normally distributed. Figure 6 presents average ROI brightness data over each experimental stage and image obstruction reduction across all methods.

## 4. Discussion

RF puncture is increasingly being used in endovascular procedures to traverse scarring, thrombus, or other dense tissues, which may fail traditional methods [4,5,6,7,8]. RF puncture vaporizes tissue in short bursts with readjustment of the wire’s trajectory in between RF applications. However, trapped microbubbles often block wire tip visualization on IVUS and prevent proper trajectory readjustment [9,10]. This study demonstrates the early feasibility of using high-MI US to disrupt RF-generated microbubbles and facilitate IVUS guidance of RF wires.

During all experimental trials, RF-generated microbubbles were visualized under IVUS guidance. These bubbles spontaneously disseminated in solution or accumulated in the model tissue. The trapped microbubbles significantly increased tissue echogenicity around the puncture trail, thereby obstructing visualization of the wire tip on IVUS guidance. Three methods to reduce RF-generated microbubble obstruction of IVUS imaging were tested: altering the MI of IVUS, VF10-5 probe application, and L12-3 probe application. Application of the L12-3 probe consistently exposed a more defined puncture trail on IVUS imaging, particularly at the start and end of the puncture trail, where bubbles tended to aggregate. When compared to the other methods, it showed the most visually significant disruption of the trapped microbubbles and overall decrease in image brightness. By reducing brightness, the echogenic RF wire tip was more easily visualized and directable through the darker tissue. Quantitative ROI analysis averaged pixel brightness around the RF puncture trails at each stage of the procedure. The relative decrease in IVUS imaging obstruction from L12-3 probe application was statistically significant when compared to negligible baseline changes.

Although conducted in an in vitro bench model, these findings suggest applicability to clinical RF puncture procedures using IVUS guidance. These techniques may be particularly useful for vessel recanalization, TIPS, and other procedures with RF puncturing of dense tissue. Clinically, RF puncture is typically performed in short, intermittent bursts with pauses for wire redirection, creating an opportunity for brief external high-MI ultrasound application without disrupting the procedural flow. The external ultrasound probes evaluated in this study are widely available in procedural suites and require no modification to existing RF or IVUS systems. These factors support the feasibility of clinical integration with further studies considering in vivo and early clinical evaluation.

### Limitations

Although the puncture trail extremities and surrounding regions showed significant brightness reduction, little change was seen directly within the puncture trail. Across all RF punctures, the central portion of the trail continued to demonstrate increased brightness regardless of external influence. Examination of the tissue showed dense thermal scarring from RF energy. The scarring likely resulted from using clinical RF generator operating parameters meant for human scar tissue or cirrhosis on unscarred, raw beef liver. Echogenic scarring from RF puncture introduced additional obstruction of IVUS guidance, confounding the evaluation of microbubble IVUS guidance obstruction. The variability in thermal scar tissue from RF energy introduced experimental uncertainty and inconsistency, as all data were measured through ROI pixel brightness. This limitation may be addressed in later studies by using cirrhotic liver tissue or quantifying pathological scarring following RF puncture.

This study was also limited by its sample size and design as an in vitro bench study. These reduce the study’s external validity and reflect its place as an initial exploration of feasibility without significant statistical power to draw definitive inferences.

## 5. Conclusions

These results show early feasibility of using high-MI US to dissipate RF-generated microbubbles within a bench model. Further testing is needed to evaluate other US probe frequencies and probe shapes that could potentially optimize microbubble disruption. Moreover, evidence is needed to show clinical practicality. However, the wide availability, low cost, and low risk of using US allow for the easy adaptability to a clinical setting. RF puncture progressively cuts through tissue in short bursts (1–3 s) of RF energy with breaks to adjust wire trajectory. Therefore, an external US application could be seamlessly added to preexisting RF puncture procedures to clear image-obstructing microbubbles between bursts if needed.

## Figures and Tables

**Table 1:** An enumerated list of equipment used in the construction of a bench setup and throughout experimentation. Equipment specifications and operational settings are included where necessary.

#	Equipment	Description
1	Siemens ACUSON P500™ Ultrasound System, Siemens Medical Solutions USA, Inc., Washington, DC, USA	- Imaging ultrasound system connected to IVUS probe and VF10-5 probe
2	Biosense Webster SOUNDSTAR^®^ eco Catheter, Biosense Webster, Irvine, CA, USA	- IVUS imaging transducer connected to ACUSON P500 system
3	Siemens VF10-5 Linear Probe, Siemens Medical Solutions USA, Inc., Washington, DC, USA	- Disrupting transducer connected to ACUSON P500 system - MI: 0.9, Frequency: 5 MHz, Tx power: 100%, Mode: B
4	Philips EPIQ 7G Ultrasound System, Philips Ultrasound, Inc., Bothell, WA, USA	- Disrupting ultrasound system connected to L12-3 probe
5	Philips L12-3 Broadband Linear Array Transducer, Philips Ultrasound, Inc., Bothell, WA, USA	- Disrupting transducer connected to EPIQ 7G system - MI: 1.2, Frequency: 3 MHz, Tx power: 100%, Mode: B
6	Baylis Medical Company RFP-100A RF Puncture Generator, Baylis Medical Company Inc., Montreal, QC, Canada	- Radiofrequency generator connected to PowerWire - System set to 25 W for a 3 s duration
7	Baylis Medical Technologies PowerWire^®^ RF Guidewire, Baylis Medical Company Inc., Montreal, QC, Canada	- Radiofrequency wire connected to RFP-100A Generator - Straight tip 50 g strength

**Table 2:** An enumerated list of materials used in the construction of a bench setup and throughout experimentation. Note: construction tools and other common materials are not included.

#	Material	Description
1	Acrylic sheets	- 0.32 cm thick acrylic sheet (~500 in^2^) - 0.64 cm thick acrylic sheet (~700 108 in^2^)
2	Acrylic glue	- SureHold plastic surgery super glue
3	Sealant	- Star brite clear marine silicone sealant
4	Tube fittings	- 2 × tube fittings with securable screw ends available at McMaster-Carr: https://www.mcmaster.com/5225K37/ (accessed on 12 January 2022). - Hole diameter = 16 mm - Tube diameter = 8 mm
5	Tube fitting connectors	- 2 × connectors which lock into tube fitting available at McMaster-Carr: - https://www.mcmaster.com/5225K174/ (accessed on 12 January 2022). - For tube D = 8 mm
6	O-rings	- 4 × lubricated black rubber O-ring - OD = 21 mm; ID = 16 mm;
7	Silicone tubing	- Silicon connection tubing (5 cm) - OD = 10 mm; ID = 5 mm
8	Tuohy-Borst adapters	- 2 × Tuohy-Borst adapters with male Luer lock available at QOSINA: https://www.qosina.com/tuohy-borst-adapter-male-luer-lock-80408?VariantID=Version_v-01 (accessed on 12 January 2022).
9	RF wire support tube	- Short, rigid plastic tubing into which RF guidewire is inserted - OD = 1 mm
10	Tissue holder	- 3d printed, gray structure to secure liver tissue submerged in saline
11	Plastic screws	- 4 × 4–40 thread black plastic screws cut to the required length
12	Aluminum foil	- Small balled up sheet of aluminum foil
13	Physiological salt solution	- 0.9% NaCl (wt.) aqueous solution made with distilled water
14	Liver tissue	- Fresh store-bought beef liver

**Table 3:** Raw ROI brightness data for baseline, IVUS MI increase, VF10-5, and L12-3 trials. Calculated L12-3 averages, brightness differences, and % reduction of RF puncture obstruction are included.

Experimental Stage	Baseline	IVUS MI Increase	VF10-5	L12-3 (1)	L12-3 (2)	L12-3 (3)	L12-3 Average
Before Puncture	0.356	0.354	0.281	0.389	0.420	0.466	0.425
After Puncture	0.577	0.543	0.381	0.532	0.628	0.681	0.614
After Application	0.580 ^a^	0.541	0.382	0.490	0.515	0.648	0.551
RF Puncture Brightness Increase	0.221	0.189	0.101	0.143	0.208	0.215	0.188
Brightness Reduction	−0.003	0.002	−0.001	0.042	0.113	0.033	0.063
% Reduction RF Puncture Obstruction	−1.5	1.2	−1.2	29.4	54.1	15.5	33.0

^a^ 2 min post-RF puncture with no external influence on the system.

## Data Availability

The original contributions presented in this study are included in the article. Further inquiries can be directed to the corresponding author.

## Abbreviations

RF	Radiofrequency wire
US	Ultrasound
IVUS	Intravascular ultrasound
MI	Mechanical index
ROI	Region of Interest
